# Commentary: Communication between Viruses Guides Lysis–Lysogeny Decisions

**DOI:** 10.3389/fmicb.2017.00983

**Published:** 2017-05-31

**Authors:** Stephen T. Abedon

**Affiliations:** Department of Microbiology, The Ohio State UniversityMansfield, OH, United States

**Keywords:** bacteriophage ecology, extracellular signaling, lysis inhibition, multiplicity of infection, phage

Communication between bacteria, via quorum sensing, has been a hot topic for some time (Miller and Bassler, 2001). Communication between bacteriophage- (phage-) infected bacteria has been much less studied, though predates the discovery of quorum sensing (Table 1), plus an offshoot played a prominent role in characterization of the fine structure of genes (Benzer, 1955). A conceptually related phenomenon has been observed with phage lambda involving lysis–lysogeny decisions. As I've had an interest in these systems for ~30 years, here I provide some historical as well as ecological perspective.

**Table 1:** Mechanisms of communication between phage-infected bacteria.

	**Lysis inhibition**	**High-multiplicity lysogeny decisions**	**Lysis-inhibition collapse**	**Arbitrium system**
References to establishing experiments	Hershey, 1946; Doermann, 1948	Boyd, 1951; Lieb, 1953; Levine, 1957; Fry, 1959; Six, 1961; Brooks, 1965; Hoffman and Rubenstein, 1968; Kourilsky, 1973	Abedon, 1992, 1999	Erez et al., 2017
Phages	T-even type (e.g., coliphages T2, T4, T6)	Temperate phages such as phage lambda	T-even type (phage T4 experiments)	*B. subtilis* phage phi3T and others
Source of signal	Lysing infections	Lysing infections	Lysing infections	Intact Infections
Inter-cellular signal	Adsorbing virions	Infecting virions	Adsorbing virions	Received peptide
Recipient	Established lytic infection	Establishing infection (pre-lysogenization)	Lysis inhibited lytic infection	Establishing infection (pre-lysogenization)
Motivator of response	Recipient of signal	Recipient of signal	Source of signal	Recipient of signal
Response	Extension of established lytic cycles	Biases lysis–lysogeny decision to lysogeny	Acceleration of population-wide lysis	Biases lysis–lysogeny decision to lysogeny
Utility (all reduce potential for progeny virion adsorption to already phage-infected bacteria)	Retention of host when phage-uninfected host bacteria are less prevalent	Retention of host when phage-uninfected host bacteria are less prevalent	Removal via coerced lysis of virion-inactivating phage-infected bacteria from environment	Retention of host when phage-uninfected host bacteria are less prevalent
Recipient gene expression	Various rapid lysis *(r)* genes, particularly *rI*; see Burch et al., 2011	Genes *cII* and possibly *cIII* in phage lambda; see Kourilsky, 1974	Recipient resistance to lysis from without via genes *imm* and *sp*	Genes *aimR* and *aimX*, note also *aimP* which encodes the signal
Ecological context	High infected-cell densities	High infected-cell densities	High infected-cell densities	High infected-cell densities

In a fascinating study, Erez et al. (2017) found that phage phi3T-infected *Bacillus subtilis* provides extracellular signals, consisting of hexapeptides, which are detected by newly phage phi3T-infected bacteria. This “arbitrium” system requires three phage genes: One produces the peptide, another serves as receptor, and the third regulates the display of lysogeny. Thus, phi3T-infected bacteria produce an extracellular signal which, if present in sufficient quantities, has the effect of increasing the likelihood of display of lysogenic cycles by newly phage-infected bacteria. If insufficient signal is present, then there is greater tendency for infections to instead display lytic cycles. Phages thus can extend their infection periods, as prophages, when potential host bacteria presumably are mostly phage infected, but exploit those bacteria lytically when neighboring bacteria are less likely to be already phage infected. Erez et al. conclude by noting that, “To our knowledge, this study is the first demonstration of actual small-molecule communication between viruses.” To my knowledge that statement is technically correct. As alluded to in the first paragraph of this commentary, however, the Erez et al. study is not the first to demonstrate communication between viruses, or more specifically between virus-infected bacteria.

The virus-to-virus communication described by Erez et al. (2017) is unidirectional, involving the release of a factor, a short peptide, which is both received by and influences the physiology of different phage-infected bacteria. That idea, however, that phage-infected bacteria can generate extracellular factors which can influence the physiology of other phage infections was, to my knowledge, first and in ways similarly presented by Doermann (1948) as a phenotype of T-even type phages; see also Hershey (1946). Here it is phage virions themselves that serve as the extracellular signal, as received in the form of secondary adsorptions (Abedon, 1994, 2015). By some as yet not fully characterized mechanism (Moussa et al., 2012), this results in an extension of the infecting phage's latent period (lytic cycle), with this extension coinciding with enhancement of the phage burst size.

The possible ecological underpinnings of the phenomenon of lysis inhibition were first pointed out and subsequently elaborated upon by myself (Abedon, 1990, 2008, 2009, 2011a, 2012). As echoed by Erez et al. (2017), “The biological logic behind this strategy is clear: when a single phage encounters a bacterial colony, there is ample prey for the progeny phages that are produced from the first cycles of infection, and hence a lytic cycle is preferred. In later stages of the infection dynamics, the number of bacterial cells is reduced to a point that progeny phages are at risk of no longer having a new host to infect.” Thus, the phages extend their infections presumably to more fully exploit increasingly rare bacterial hosts, whether using lysogenic cycles or, instead, via lysis inhibition.

A second example of communication between phage infections was also noted, by myself, within the context of lysis inhibition (Abedon, 1992). Lysis inhibited bacteria face a dilemma as a consequence of lysis inhibition (Abedon, 2008, 2009), and this stems from a display of superinfection exclusion by phage-infected bacteria (Abedon, 1994). In a population of lysis-inhibited bacteria, the first infections to lyse will expose their virion progeny to already phage-infected bacteria. Sufficiently high local densities of these phage-infected bacteria can result in rapid inactivation of those virions, i.e., as due to superinfection exclusion. A solution to this problem is to wait, via continued lysis inhibition, until the rest of the phage population has lysed before releasing phage progeny. If all local infections were to so wait, however, then the expectation would be that lysis would never occur and thereby no disseminating virions would be released to locate new hosts, hence the dilemma. One solution is for infections to lyse more or less simultaneously, which in the laboratory turns out to be just what they do. The signal that conveys this coordination between otherwise independent bacteria is supplied by other infections, again in the form of virions. The mechanism itself appears to resemble a phenomenon known as lysis from without (Abedon, 1992, 1999, 2011b).

Lysis inhibition represents a conditional increase in a phage's infection period in association with an increase in a phage's burst size. Lysogeny represents a conditional increase in a phage's infection period in association, at least potentially, with an increase in the *number* of phage bursts (Abedon, 2008, 2009). As Erez et al. note, the decision to enter lysogenic cycles can be influenced by secondary adsorptions, or more specifically in this case, by multiple infection of otherwise uninfected bacteria. Thus, just as with lysis inhibition, when multiple phages which are able to adsorb individual bacteria are present within an environment, then this has the effect of inducing extensions in latent periods, that is, biasing infections toward lysogenic cycles (e.g., see Weitz et al., 2008).

Erez et al. (2017) found that signals provided by predecessor infections can influence the behavior of subsequent infections, changing the behavior of the newer infections in response to the existence of high local densities of phage-infected bacteria. As noted, at least three instances have already been described of similar communication between phage infections, each also serving to mitigate issues associated with phage-infection “overcrowding.” These other mechanisms all employ whole phage virions as the signal. An important ecological question therefore is why employ peptide-based lytic cycle-suppression given use, toward similar ends, of virion-mediated communication by other phages? Perhaps to achieve redundant, sooner, or additive activity? Origination of signals also from already established lysogens is another possibility (Hynes and Moineau, 2017).

## Author contributions

The author confirms being the sole contributor of this work and approved it for publication.

### Conflict of interest statement

The author declares that the research was conducted in the absence of any commercial or financial relationships that could be construed as a potential conflict of interest.

## References

[B1] Abedon S. T. (1990). Selection for lysis inhibition in bacteriophage. J. Theor. Biol. 146, 501–511. 10.1016/S0022-5193(05)80375-3 2273898

[B2] Abedon S. T. (1992). Lysis of lysis inhibited bacteriophage T4-infected cells. J. Bacteriol. 174, 8073–8080. 10.1128/jb.174.24.8073-8080.1992 1459956 PMC207546

[B3] Abedon S. T. (1994). Lysis and the interaction between free phages and infected cells, in The Molecular Biology of Bacteriophage T4, eds Karam J. D. Kutter E. Carlson K. Guttman B. (Washington, DC: ASM Press), 397–405.

[B4] Abedon S. T. (1999). Bacteriophage T4 resistance to lysis-inhibition collapse. Genet. Res. 74, 1–11. 10.1017/S0016672399003833 10505404

[B5] Abedon S. T. (2008). Phage population growth: constraints, games, adaptation, in Bacteriophage Ecology, ed Abedon S. T. (Cambridge, UK: Cambridge University Press), 64–93. 10.1017/cbo9780511541483.006

[B6] Abedon S. T. (2009). Bacteriophage intraspecific cooperation and defection, in Contemporary Trends in Bacteriophage Research, ed Adams H. T. (Hauppauge, NY: Nova Science Publishers), 191–215.

[B7] Abedon S. T. (2011a). Bacteriophages and Biofilms: Ecology, Phage Therapy, Plaques. Hauppauge, NY: Nova Science Publishers.

[B8] Abedon S. T. (2011b). Lysis from without. Bacteriophage 1, 46–49. 10.4161/bact.1.1.13980 21687534 PMC3109453

[B9] Abedon S. T. (2012). Thinking about microcolonies as phage targets. Bacteriophage 2, 200–204. 10.4161/bact.22444 23275871 PMC3530530

[B10] Abedon S. T. (2015). Bacteriophage secondary infection. Virol. Sin. 30, 3–10. 10.1007/s12250-014-3547-2 25595214 PMC8200926

[B11] Benzer S. (1955). Fine structure of a genetic region in bacteriophage. Proc. Natl. Acad. Sci. U.S.A. 41, 344–354. 10.1073/pnas.41.6.344 16589677 PMC528093

[B12] Boyd J. S. (1951). Observations on the relationship of symbiotic and lytic bacteriophage. J. Pathol. Bacteriol. 63, 445–457. 10.1002/path.1700630311 14874159

[B13] Brooks K. (1965). Studies in the physiological genetics of some supporessor-sensitive mutants of bacteriophages lambda. Virology 26, 489–499. 10.1016/0042-6822(65)90011-5 14319719

[B14] Burch L. H. Zhang L. Chao F. G. Xu H. Drake J. W. (2011). The bacteriophage T4 rapid-lysis genes and their mutational proclivities. J. Bacteriol. 193, 3537–3545. 10.1128/JB.00138-11 21571993 PMC3133318

[B15] Doermann A. H. (1948). Lysis and lysis inhibition with *Escherichia coli* bacteriophage. J. Bacteriol. 55, 257–275. 16561455 10.1128/jb.55.2.257-276.1948 PMC518436

[B16] Erez Z. Steinberger-Levy I. Shamir M. Doron S. Stokar-Avihail A. Peleg Y.. (2017). Communication between viruses guides lysis-lysogeny decisions. Nature 541, 488–493. 10.1038/nature21049 28099413 PMC5378303

[B17] Fry B. A. (1959). Conditions for the infection of *Escherichia coli* with lambda phage and for the establishment of lysogeny. J. Gen. Microbiol. 21, 676–684. 10.1099/00221287-21-3-676 13825457

[B18] Hershey A. D. (1946). Mutation of bacteriophage with respect to type of plaque. Genetics 31, 620–640. 17247224 10.1093/genetics/31.6.620 PMC1209358

[B19] Hoffman D. B. Jr. Rubenstein I. (1968). Physical studies of lysogeny. I. Properties of intracellular parental bacteriophage DNA from λ*-infected sensitive bacteria*. J. Mol. Biol. 35, 375–399. 10.1016/S0022-2836(68)80001-4 4877000

[B20] Hynes A. P. Moineau S. (2017). Phagebook: the social network. Mol. Cell 65, 963–964. 10.1016/j.molcel.2017.02.028 28306511

[B21] Kourilsky P. (1973). Lysogenization by bacteriophage lambda. I. Multiple infection and the lysogenic response. Mol. Gen. Genet. 122, 183–195. 10.1007/BF00435190 4573866

[B22] Kourilsky P. (1974). Lysogenization by bacteriophage lambda. II. Identification of genes involved in the multiplicity dependent processes. Biochimie 56, 1511–1516. 10.1016/S0300-9084(75)80274-4 4619341

[B23] Levine M. (1957). Mutations in the temperate phage P22 and lysogeny in Salmonella. Virology 3, 22–41. 10.1016/0042-6822(57)90021-1 13409758

[B24] Lieb M. (1953). The establishment of lysogenicity in *Escherichia coli*. J. Bacteriol. 65, 642–651. 13069436 10.1128/jb.65.6.642-651.1953 PMC169593

[B25] Miller M. B. Bassler B. L. (2001). Quorum sensing in bacteria. Annu. Rev. Microbiol. 55, 165–199. 10.1146/annurev.micro.55.1.165 11544353

[B26] Moussa S. H. Kuznetsov V. Tran T. A. Sacchettini J. C. Young R. (2012). Protein determinants of phage T4 lysis inhibition. Protein Sci. 21, 571–582. 10.1002/pro.2042 22389108 PMC3375757

[B27] Six E. (1961). Inheritance of prophage P2 in superinfection experiments. Virology 14, 220–233. 10.1016/0042-6822(61)90197-0

[B28] Weitz J. S. Mileyko Y. Joh R. I. Voit E. O. (2008). Collective decision making in bacterial viruses. Biophys. J. 95, 2673–2680. 10.1529/biophysj.108.133694 18567629 PMC2527279

